# Lithiophilic Interlayer with Electrolyte-Reservoir and Dendrite-Buffer for High-Performance Lithium Metal Batteries

**DOI:** 10.3390/nano15100710

**Published:** 2025-05-09

**Authors:** Huasen Shen, Guoning Wu, Tingting Ma, Mengjun Li, Yunan Tian, Si Chen, Shaojun Cai, Zhaohuai Li

**Affiliations:** 1 Key Laboratory of Flexible Optoelectronic Materials and Technology, Ministry of Education, Jianghan University, Wuhan 430056, China; hssdsb@stu.jhun.edu.cn (H.S.); guoningwu@stu.jhun.edu.cn (G.W.); tingtingma@stu.jhun.edu.cn (T.M.); mengjunli@stu.jhun.edu.cn (M.L.); tianyunan@stu.jhun.edu.cn (Y.T.); s.chen@stu.jhun.edu.cn (S.C.); 2 Hubei Provincial Engineering Research Center of Surface and Interface Regulation Technology and Equipment for Renewable Energy Materials, Jianghan University, Wuhan 430056, China; 3 School of Optoelectronic Materials and Technology, Jianghan University, Wuhan 430056, China

**Keywords:** lithium metal anodes (LMAs), dendrite, atomic layer deposition (ALD), carbon nanotube (CNT), lithiophilic interlayer

## Abstract

Uneven local electric fields and limited nucleation sites at the reaction interface can lead to the formation of hazardous lithium (Li) dendrites, posing a significant safety risk and impeding the practical utilization of Li metal anodes (LMAs). Here, we present a method utilizing atomic layer deposition (ALD) to create lithiophilic titanium nitride (TiN) sites on carbon nanotubes (CNTs) surfaces, integrated with nanocellulose to form a lithiophilic interlayer (NFCP@TN). This interlayer, which is highly flexible and electrolyte-wettable, functions as a current collector and host material for LMAs. The uniform deposition of Li is facilitated by the synergistic interplay of the lithiophilic active sites TiN, the conductive CNT network, and excellent electrolyte wettability of nanocellulose. As a result, Li preferentially adsorbs on TiN sheaths with lower diffusion barriers, leading to controlled nucleation sites and dendrite-free Li deposition. Furthermore, the well-designed NFCP@TN interlayer exhibits exceptional electrochemical performance and significantly extended cycle life when paired LMA with high areal capacity NCM811 (5.0 mAh cm^−2^) electrodes.

## 1. Introduction

Amid the global shift towards sustainable energy, the advancement of energy storage technology stands as a pivotal driver. Lithium (Li)-ion batteries (LIBs), as a prevalent energy storage solution, find widespread application in portable electronics, electric vehicles, and large-scale energy storage systems, fundamentally altering energy consumption patterns [1,2]. However, with technological progress and expanding application domains, the energy density of conventional LIBs with intercalated anodes is increasingly inadequate to meet the escalating demand for high energy storage capacity [3,4,5]. Consequently, researchers are actively exploring new battery systems, with Li metal anodes (LMAs) emerging prominently due to their exceptional theoretical specific capacity (3860 mAh g^−1^) and remarkably low redox potential (−3.04 V vs. standard hydrogen electrodes) [6,7].

The evolution of Li-metal batteries (LMBs) traces back to their nascent exploration phase, where researchers recognized their substantial potential for enhancing battery energy density. However, on the path to practical implementation, LMAs have encountered a series of formidable challenges akin to formidable barriers, severely impeding their commercialization progress. During charge–discharge cycles, LMA exhibits uneven deposition, giving rise to the formation of Li dendrites. The presence of these dendrites not only markedly diminishes the battery’s Coulombic efficiency, leading to a rapid capacity decline, but more critically, they can breach the separator, causing internal short-circuiting and triggering a cascade of severe safety hazards such as thermal runaway, fire, explosions, and other catastrophic outcomes, posing a significant threat to battery safety and lifespan [8,9]. Concurrently, the coexistence of LMAs and the electrolyte fosters aggressive side reactions, resulting in the generation of an unstable solid electrolyte interface (SEI) layer on the LMA surface. This SEI layer undergoes continuous growth and rupture throughout battery cycling, depleting both LMA and electrolyte, thereby deteriorating battery performance, shortening battery lifespan [10].

Despite the formidable challenges, recent years have witnessed significant breakthroughs in LMA modifications, owing to profound interdisciplinary collaborations and innovative strides in materials science, nanotechnology, and electrochemistry [11]. In terms of electrode structure design, the construction of three-dimensional (3D) porous LMAs has emerged as an effective strategy [12]. This unique structural configuration markedly diminishes local current density, furnishes ample active sites for Li deposition, suppresses dendrite growth at its inception, greatly enhances LMA cycling stability, and opens avenues for prolonged battery operation [13,14]. Atomic Layer Deposition (ALD) technology enables the creation of nanoscale lithiophilic sites, crucial for optimizing Li nucleation on an atomic scale. Carbon materials, boasting abundant resources and exceptional conductivity, play a pivotal role in Li^+^ conduction and Li growth, garnering widespread favor among researchers delving into LMBs [15,16,17,18]. Nanocellulose, rich in hydroxyl groups on its surface, exhibits robust wettability and liquid retention capabilities, acting as an electrolyte reservoir. Moreover, the high strength and Young’s modulus of nanocellulose fortify the mechanical properties of composite anodes, enabling them to accommodate the volume fluctuations during cyclic Li plating and stripping.

In this study, ALD technology is employed to fabricate a highly flexible TiN-modified lithiophilic interlayer on carbon nanotubes (CNTs@TN). This approach is specifically tailored to conduct an in-depth systematic investigation of Li nucleation and to meticulously analyze the critical safety concerns stemming from the current challenges posed by Li dendrite growth. By leveraging the inherent affinity of Li for TiN and its enhanced diffusion properties, CNTs are intricately integrated into a highly interconnected conductive network, ensuring swift Li growth. Additionally, the strong flexibility and electrolyte wettability of nanocellulose are harnessed to fortify the interlayer, enabling it to endure prolonged cycles of Li electroplating and stripping. As results, when integrated into a full cell configuration with nanocellulose CNTs@TN paper (NFCP@TN) serving as the interlayer paired with high areal capacity cathode NCM811 (5.0 mAh cm^−2^), the LMB demonstrates stable cycling over 100 cycles, a performance nearly seven times superior to that of nanocellulose carbon paper (NFCP) alone. This outcome underscores the substantial potential of NFCP@TN for the practical implementation of LMBs.

## 2. Results and Discussion

**Figure 1:**
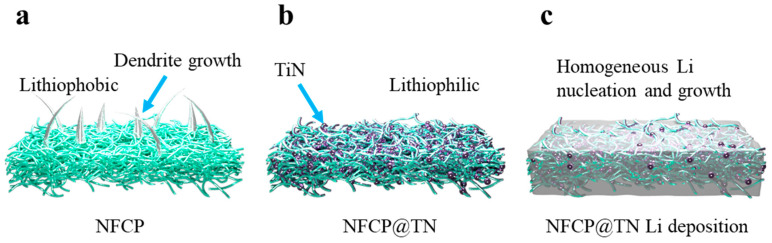
The schematic of Li deposition on (**a**) NFCP, the structure of (**b**) NFCP@TN and (**c**) NFCP@TN after Li deposition.

NFCP in conjunction with nanocellulose and carbon nanotubes exhibits a substantial specific surface area and appropriate porosity, rendering it an optimal structure for Li nucleation growth. However, the absence of lithiophilic sites within this configuration results in a stochastic intrinsic nature of Li nucleation growth on its surface, leading to the formation of needle-like Li dendrites (Figure 1a). These dendrites pose significant safety risks in the application of LMBs due to potential internal short-circuiting hazards. In contrast to NFCP, the NFCP (Figure 1b) with pronounced lithiophilic properties incorporates TiN, Li-friendly sites on CNTs engineered using ALD technology (Supplementary Figures S1 and S2). This structure effectively diminishes local current densities, furnishing numerous nucleation sites for Li deposition, it is notable that the porosity of NFCP and NFCP@TN was 52% and 51%, respectively, which means that the construction of lithiophilic using ALD technology does not lose the volumetric energy density of the interlayer. Furthermore, the excellent electrolyte wettability of nanocellulose enables the structure to function as an ‘electrolyte reservoir’, facilitating rapid Li^+^ transfer and ensuring a uniform and stable Li deposition devoid of dendritic growth (Figure 1c).

**Figure 2:**
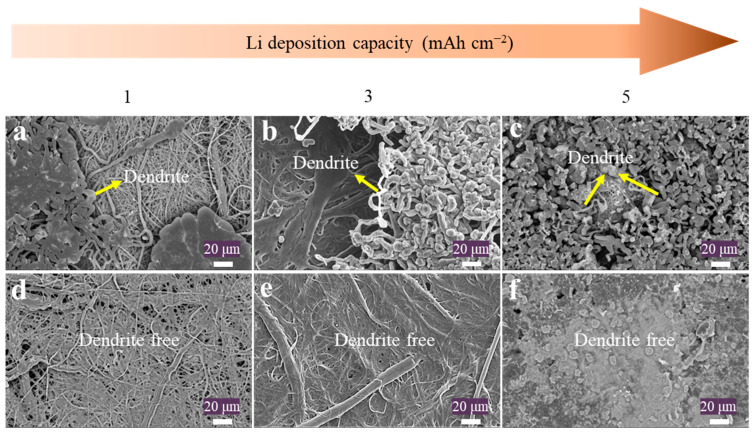
SEM images of Li deposition on NFCP and NFCP@TN with (**a**,**d**) 1, (**b**,**e**) 3, and (**c**,**f**) 5 mAh cm^−2^.

To ascertain the efficacy of NFCP@TN in suppressing Li dendrites, experiments involving varying Li deposition capacities were conducted on NFCP and NFCP@TN at a current density of 1 mA cm^−2^. The scanning electron microscopy (SEM) images reveal pronounced Li aggregation and localized growth on the surface of NFCP during Li plating (Figure 2a,b), with needle-like Li dendrites emerging when the Li deposition capacity reached 5 mAh cm^−2^ (Figure 2c). Conversely, Li nucleation and deposition on NFCP@TN exhibited remarkable uniformity (Figure 2d,e). As the Li plating capacity increased to 5 mAh cm^−2^ (Figure 2f), NFCP@TN fibers became progressively enveloped by a uniform Li metal plating layer, with the inter-fiber pores filled with deposited Li. Morphological observation of the cross-section of the collector after Li deposition reveals that severe Li dendrite growth accompanies the NFCP, and the Li dendrite growth is more severe with the increase in Li deposition capacity (Supplementary Figure S3a,b). On the contrary, NFCP@TN demonstrates obvious Li dendrite inhibition after Li deposition, and with the increase in Li deposition capacity, NFCP@TN further exhibits the ability to accommodate Li metal, which is inextricably linked to our proposed mechanism of utilizing TiN lithiophilic sites to improve Li metal deposition behavior (Supplementary Figure S3c,d). In order to investigate the effect of TiN on the Li nucleation mechanism, the initial deposition process of Li on NFCP@TN is observed (Supplementary Figure S4), and it can be found that, under the influence of the adsorption of TiN, Li is first nucleated on the surface of CNTs, providing a smooth surface for the subsequent uniform deposition of Li metal.

**Figure 3:**
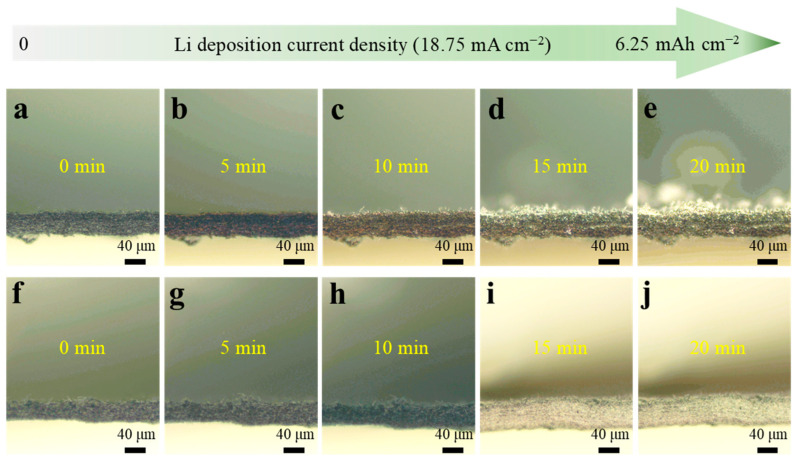
In situ microscopy images of Li deposition behaviors on (**a**–**e**) NFCP and (**f**–**j**) NFCP@TN with various stages.

Furthermore, in situ microscopy was employed to observe Li metal deposition behavior on NFCP, Cu and NFCP@TN, with a current density of 18.75 mA cm^−2^, electrodeposition time of 20 min, and Li deposition capacity of 6.25 mAh cm^−2^. Obviously, the Li dendrite growth on NFCP and Cu commenced at 10 min and intensified with prolonged electrodeposition time (Figure 3a–e, Supplementary Figure S5). In contrast, NFCP@TN demonstrated significant suppression of Li dendrites throughout the electrodeposition process, effectively modulating Li deposition behavior. The Li metal grew uniformly on the surface of NFCP@TN, with the desired Li metal deposition morphology clearly visible at 15 and 20 min of electrodeposition (Figure 3f–j). The observation of Li deposition behavior on NFCP@TN highlights the efficacy of TiN in regulating Li deposition behavior and suppressing Li dendritic growth. These observations suggest that uncontrolled Li nucleation and random Li plating on NFCP lead to the formation of dendritic and dead Li, diminishing Li utilization and compromising cycling stability. On the contrary, the combination of NFCP with highly lithiophilic sites (TiN) and excellent wetting properties towards the electrolyte enhances uniform Li nucleation and accelerates the planar diffusion of deposited Li, facilitating the dendrite-free growth of Li metal at the anode interface.

**Figure 4:**
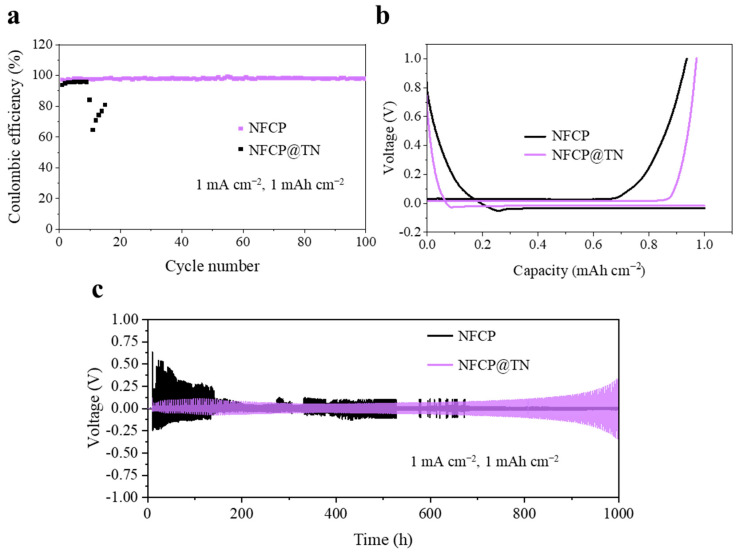
(**a**) CE comparison of NFCP and NFCP@TN current collectors at current of 1 mA cm^−2^ with areal capacity of 1 mAh cm^−2^. (**b**) Corresponding voltage profiles of NFCP and NFCP@TN current collectors at 1 mA cm^−2^ with areal capacity of 1 mAh cm^−2^. (**c**) Voltage profiles of symmetric NFCP@Li and NFCP@TN@Li cells at 3 mA cm^−2^ for 3 mAh cm^−2^.

To evaluate the application potential of NFCP@TN in LMBs, Coulombic efficiency (CE) tests were conducted to investigate the impact of NFCP and NFCP@TN interlayers on LMAs performance (Figure 4a). Half-cells utilizing NFCP or NFCP@TN as the working electrode and Li metal as the counter electrode were employed to assess CE. At a current density of 1 mA cm^−2^ and a Li plating/stripping capacity of 1 mAh cm^−2^, NFCP demonstrated a significant decline in CE and reduced cycling stability after 15 cycles, indicating the formation of numerous Li dendrites and dead Li. This observation aligns with the in situ microscopy findings, indicating that the repeated growth of Li dendrites on the NFCP surface negatively impacts LMAs utilization. In contrast, NFCP@TN exhibited enhanced CE stability and an extended cycle life, attributed to the facilitation of Li metal nucleation and growth by TiN lithiophilic sites, effectively suppressing the Li dendrite formation and with a stable SEI (Supplementary Figure S6). The corresponding charge/discharge curves (Figure 4b) exhibit remarkable stability and minimal overpotentials, likely due to the presence of TiN nanoparticles and the intricate 3D structure facilitating the uniform deposition of Li metal with exceptional reversibility.

Moreover, Li||Li symmetric cells were further constructed to investigate the Li plating/stripping behavior. The symmetric cell incorporating NFCP@TN@Li electrodes demonstrated a consistently low overpotential during cycling, with a noticeable increase observed after exceeding 800 h at 3 mA cm^−2^ and 3 mAh cm^−2^ per cycle. This behavior can be ascribed to the superior Li^+^ conductivity facilitated by the TiN active sites and the well-designed structure, which enhance rapid Li^+^ migration and diffusion throughout the whole interphase. However, the symmetric cells utilizing NFCP@Li electrodes exhibited significant overpotential fluctuations after 130 h. Additionally, the symmetric NFCP@TN@Li cell showcased a minimal voltage hysteresis of approximately 102 mV, outperforming the NFCP@Li cell (307 mV) (Figure 4c). These outcomes are attributed to the uniform Li deposition sites of NFCP@TN, coupled with its exceptional electrolyte retention capabilities, which suppress Li dendrite growth, stabilize Li^+^ transport at the interface, and enhance the stability of the interfacial interlayer, thereby bolstering the cycling lifespan of the LMA.

**Figure 5:**
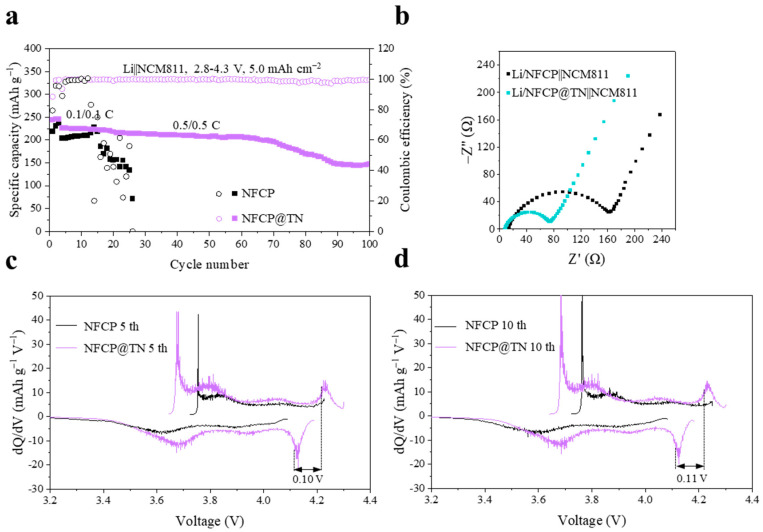
(**a**) Cycling performance of charge/discharge at 0.5 C, (**b**) EIS before cycling of the Li/NFCP||NCM811, Li/NFCP@TN||NCM811 full cells. The corresponding dQ dV^−1^ curves of the Li/NFCP||NCM811, Li/NFCP@TN||NCM811 full cells after (**c**) 5, (**d**) 10 cycles at 0.5 C.

To further exemplify the potential of NFCP@TN for practical applications, a Li/NFCP@TN||NCM811 full cell was assembled, utilizing a high areal loading cathode NCM811 (5 mAh cm^−2^), resulting in a notable enhancement in cycle life at 0.5 C. The stark contrast in the abnormal capacity retention of the Li/NFCP||NCM811 cell is noteworthy, as it exhibited significant instability in cycling after 12 cycles, with the retention plummeting to 47.3% after 25 cycles (Figure 5a). Subsequent SEM analysis unveiled substantial dendrite growth on the surface of the NFCP in the discharged state of the full cell after 25 cycles, potentially contributing to the degradation of Li/NFCP||NCM811 cell cycling performance (Supplementary Figure S7). SEM analysis of the NFCP exhibited a substantial accumulation of inactive Li on its top surface (Supplementary Figure S8). In contrast, the surface of NFCP@TN remained unblemished post-cycling, devoid of noticeable dendrite formations (Supplementary Figures S9 and S10), along with its rapid ionic conductivity (Figure 5b), suggest that TiN effectively lowers the nucleation barrier of Li^+^, while the superior electrolyte wettability and liquid retention capacity of nanocellulose further enhance performance. Furthermore, the Li/NFCP||NCM811 full cell exhibits severe ionic hindrance and severe voltage polarization during cycling, due to the stable SEI formed on NFCP@TN after lithiation of the lithiophilic sites TiN, so the Li/NFCP@TN||NCM811 full cell exhibits less voltage polarization during cycling (Figure 5c,d). These electrochemical test results further demonstrate the promising prospects of NFCP@TN in practical applications within LMBs. Simultaneously, they also offer a new direction for the design of lightweight Li anode interlayers and high-capacity LMBs.

## 3. Conclusions

In summary, the combination of ALD and nanocellulose was utilized to create 3D integrated lithophilic structures (NFCP@TN), effectively guiding Li deposition and suppressing dendritic growth. The incorporation of TiN and nanocellulose architectures on the NFCP@TN scaffold reduced the nucleation barrier for Li and alleviated volume fluctuations during cycling. The TiN active sites promoted uniform Li nucleation, while the resulting inorganic-rich SEI layer facilitated rapid Li^+^ diffusion, enabling dense and uniform Li deposition. Consequently, the distinctive NFCP@TN interlayer structure exhibited a strong Li affinity and minimal nucleation overpotential. Moreover, this framework demonstrated prolonged operational durability exceesding 800 h with efficient mass transport kinetics at 3 mA cm^−2^ and 3 mAh cm^−2^. Coupled with the high-capacity cathode NCM811 (5.0 mAh cm^−2^), the full cell displayed exceptional cycling stability and performance versatility, positioning it as a promising candidate for practical applications.

## Data Availability

The data that support the findings of this study are available from the corresponding author upon reasonable request.

## Supplementary Materials

The following supporting information can be downloaded at: https://www.mdpi.com/article/10.3390/nano15100710/s1, Figure S1: Elemental mapping images for (b) carbon, (c) nitrogen and (d) titanium of CNT@TiN; Figure S2: The XPS fine spectra of N 1s of CNT@TiN; Figure S3: The cross-sectional SEM images of NFCP and NFCP@TN after (a,c) 1, (b,d) 3 mAh cm^−2^ Li deposition at a current density of 1 mA cm^−2^ are shown; Figure S4: The top view of NFCP@TN after 0.1 mAh cm^−2^ Li deposition; Figure S5: In situ microscopy images of Li deposition behaviors on Cu with various stages; Figure S6: The XPS spectra of NFCP and NFCP@TN electrodes for the (a) C 1s spectra, (b) F 1s spectra after 0.5 mAh cm^−2^ Li deposition at current density of 0.1 mA cm^−2^; Figure S7: The top view of NFCP after Li/NFCP||NCM811 full cell cycled. Figure S8: The cross-section of NFCP after Li/NFCP||NCM811 full cell cycled; Figure S9: The top view of NFCP@TN after Li/NFCP@TN||NCM811 full cell cycled; Figure S10: The cross-section of NFCP@TN after Li/NFCP@TN||NCM811 full cell cycled.
