# Supplementary material for: Lithiophilic Interlayer with Electrolyte-Reservoir and Dendrite-Buffer for High-Performance Lithium Metal Batteries
Source: Nanomaterials (Basel). 2025 May 9;15(10):710. doi: 10.3390/nano15100710 (PMC12113982; doi:10.3390/nano15100710)
Supplement: Supplementary file 1 [file nanomaterials-15-00710-s001.zip › nanomaterials-3557464-supplementary.pdf]

Supporting Information

Lithiophilic Interlayer with Electrolyte-Reservoir and Dendrite-Buffer for High-Performance Lithium Metal Batteries

Huasen Shen,[1,2,†] Guoning Wu,[1,†] Tingting Ma,[1,†] Mengjun Li,[1,2] Yunan Tian,[1,2] Si Chen,[1] Shaojun Cai,[3,*] Zhaohuai Li[1,2,*]

[1] Key Laboratory of Flexible Optoelectronic Materials and Technology, Ministry of Education, Jianghan University, Wuhan 430056, China.

[2] Hubei Provincial Engineering Research Center of Surface and Interface Regulation Technology and Equipment for Renewable Energy Materials, Jianghan University, Wuhan 430056, China.

[3] School of Optoelectronic Materials and Technology, Jianghan University, Wuhan 430056, China.

E-mail: shaojuncai@jhun.edu.cn (S.C.); lzhdubu@jhun.edu.cn (Z.L.)

[†]These authors contributed equally to this work.

Experimental section

Materials

Carbon nanotubes (CNTs) were acquired from M-Grade MWNT's, and the number is NTL-12112. Nanocellulose was obtained from EneRol Nanotechnologies, Inc., Ningbo. Anhydrous ethanol (> 99.8%) was purchased from Xin Shen Shi corporation (Wuhan). Atomic layer deposition (ALD) equipment was from Yun Mao technology corporation (Xiamen), and ALD technique was provided by BattFlex corporation (Wuhan). Li foil were purchased from China Energy Lithium Co., Ltd. Separator used was Celgard 2400 PP film. Polyvinylidene difluoride (PVDF), N-Methyl pyrrolidone (NMP), the solvent, 1,2-dimethoxyethane (DME), dioxolane (DOL), ethylene carbonate (EC), dimethyl carbonate (DMC), vinylene carbonate (VC), diethyl carbonate (DEC), ethyl methyl carbonate (EMC), fluoroethylene carbonate (FEC), lithium hexafluorophosphate ($LiPF_6$), lithium bis(trifluoromethanesulphonyl)imide (LiTFSI), and lithium nitrate ($LiNO_3$) were purchased from Sigma-Aldrich without further purification. Super P was purchased from AkzoNobel corporation. NCM811 ($Li[Ni_{0.8}Co_{0.1}Mn_{0.1}]O_2$) cathode materials were obtained from Rong Bai technology corporation.

NFCP@TN preparation

After a 12-hour drying process, the CNTs were coated with a 5 nm thick TiN layer using ALD technology, resulting in the formation of CNT@TiN. Before the deposition process, there are several experimental procedures we had to do with the reaction chamber: it was evacuated and filled with argon for cleaning at first, and then ozone was introduced under vacuum conditions to create a large number of defects on CNTs surfaces, followed by the re-evacuation and re-fill of argon for cleaning again. During the deposition process, $TiCl_4$ and NH_3 as the precursors and N_2 as carrier gas. Repeated above ALD steps to obtain the ideal TiN deposition, when the above cavity is fed, it is divided into three small reach pressures of 6–7 Torr, and the reaction is finally carried out under the condition of 20 Torr, all above reaction the temperatures are 450 ℃. According to areal density 1 mg cm^{-2}, CNT@TiN were mixed with nanocellulose in anhydrous ethanol solvent with the mass ratio of 8:2. To ensure complete dispersion of

the CNT@TiN, the mixture was subjected to ultrasonic vibration for 30 minutes. Subsequently, the uniformly dispersed mixture was filtered on a 500-mesh cloth to obtain the NFCP@TN paper with dimensions of 13 cm × 13 cm. After drying at 80 °C, the NFCP@TN paper was stripped from the carbon cloth. Last but not least, the NFCP@TN was further dried at 100 °C in a vacuum oven for 12 hours.

Assembly of cells

The electrochemical performance of the NFCP@TN was tested in stainless-steel cells assembled in an argon gas-filled glovebox (MIKROUNA). All the Li||CP, Li|| NFCP@TN half cells, Li/NFCP||NFCP/Li, Li/NFCP@TN||NFCP@TN/Li symmetric cells, Li/NFCP||NCM811, Li/NFCP@TN/NCM811 full cells were assembled in CR2032 coin cells. For Li||NFCP, Li||NFCP@TN half-cells, the diameter of Li anode slices is 15.8 mm and the thickness is 100 μm, while the diameter of NFCP, NFCP@TN and cathode slices is 12 mm. For Li/NFCP||NFCP/Li, Li/NFCP@TN||NFCP@TN/Li symmetric cells, the diameter of Li anode is 15.8 mm, while those of NFCP and NFCP@TN are 16 mm. For Li/NFCP||NCM811, Li/NFCP@TN||NCM811 full cells, the diameters of NCM811 cathode are 12 mm, and those of NFCP and NFCP@TN are 16 mm. 50 μL of DME/DOL (1:1, v/v) electrolyte containing 1 M LiTFSI and 2 wt% $LiNO_3$ was added to both Li||NFCP, Li||NFCP@TN half-cells and Li/NFCP||NFCP/Li, Li/NFCP@TN||NFCP@TN/Li symmetric cells.

In-situ microscopy: The dimensions of the Li, NFCP, and NFCP@TN electrodes in the Li||NFCP and Li||NFCP@TN half-cell in-situ pool were all 0.2 cm × 0.4 cm. Commercial Li discs with a diameter of 15.8 mm and a thickness of 100 μm were used. A total of 4 mL of DME/DOL (1:1, v/v) electrolyte containing 1 M LiTFSI and 2 wt% $LiNO_3$ was added to both Li||NFCP and Li||NFCP@TN half-cells. For the half-cells, in-situ microscopy imaging technique was employed under the conditions of a current density of 18.75 mA cm^{-2} and a capacity of 6.25 mAh cm^{-2}.

In Li||NCM811 full-cells, the cathode mixture comprised NCM811 (96.5 wt%), super P (1.0 wt%), PVDF (2.0 wt%), and multi-wall carbon nanotubes (MWCNTs, 0.5 wt%). The NCM811 active material loading was approximately 20 mg cm^{-2}. For each

cell, 50 μL of electrolyte composed of a mixed solvent EC/DMC (1:1, v/v), 1 M $LiPF_6$, and 2.0 wt% VC was added.

Electrochemical tests

For the Coulombic efficiency (CE) tests of Li||NFCP, Li||NFCP@TN half-cells, three cycles of pre-cycling were applied under 0.1 mA cm^{-2} and 1 mAh cm^{-2} for SEI construction. Li/NFCP||NFCP/Li and Li/NFCP@TN||NFCP@TN/Li symmetric cells were tested under 3 mAh cm^{-2} areal capacities and 3 mA cm^{-2} current densities. The cycle performance of Li||NCM811 full-cells was evaluated within a voltage range of 2.8–4.3 V and a charge/discharge rate of 0.5 C.

Structural/Physicochemical Characterization

Scanning electron microscopy (SEM) images were acquired using the JEOL JSM-7100F and Hitachi SU-8010 electron microscopes. In situ microimaging was performed using an in-situ characterization device provided by Beijing Zhongyan Huanke Science & Technology Corporation.

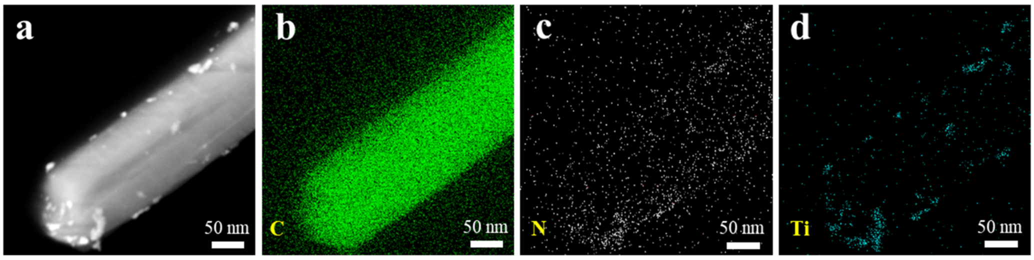

Figure S1. The TEM (a) and elemental mapping images for (b) carbon, (c) nitrogen and (d) titanium of CNT@TiN.

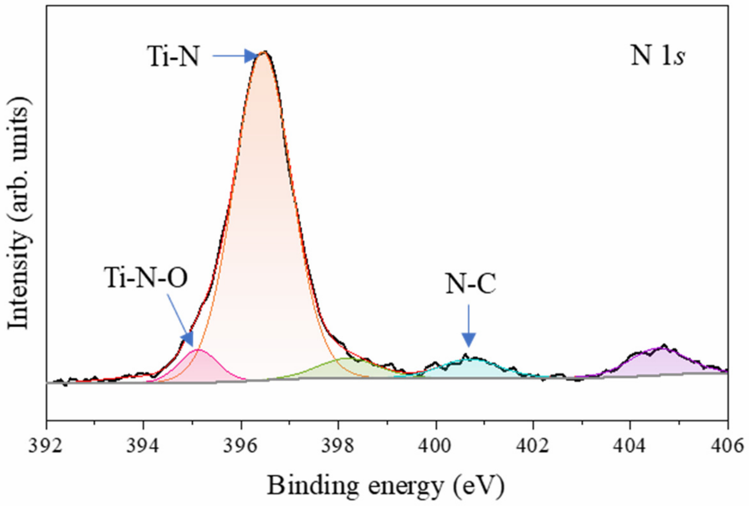

Figure S2. The XPS fine spectra of N 1*s* of CNT@TiN.

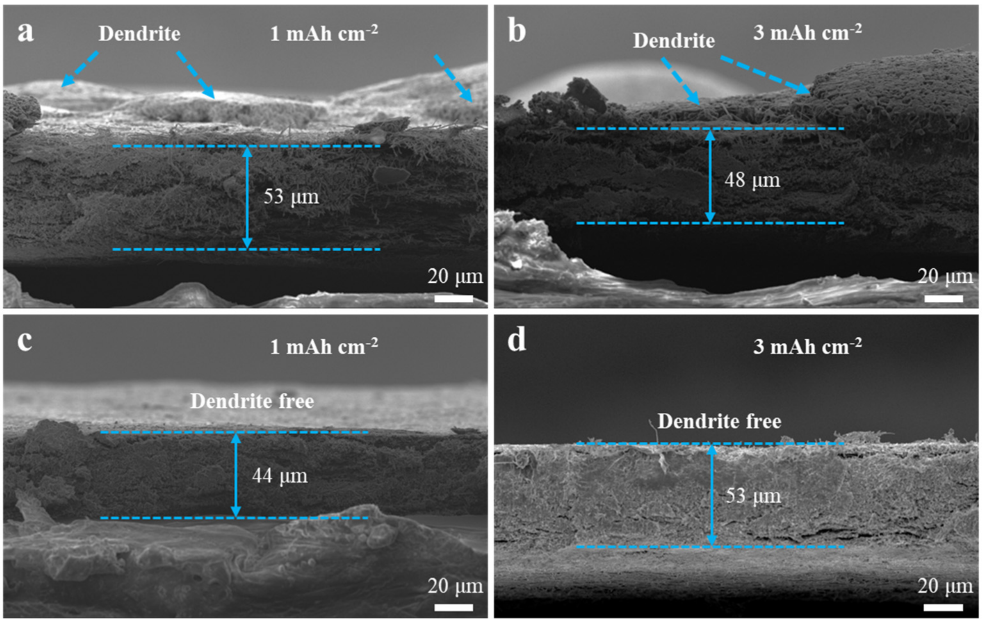

Figure S3. The cross-sectional SEM images of NFCP and NFCP@TN after (a,c) 1, (b,d) 3 mAh cm^{-2} Li deposition at a current density of 1 mA cm^{-2} are shown.

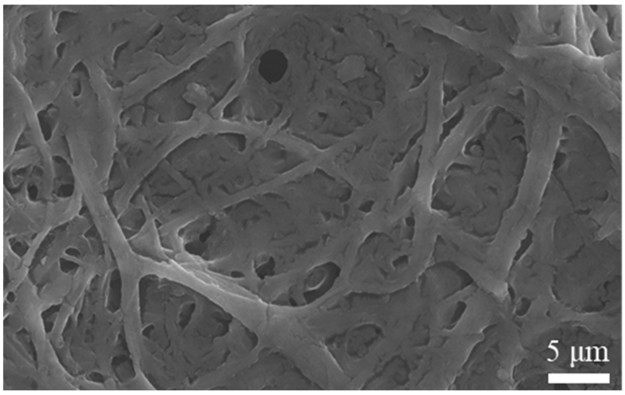

Figure S4. The top view of NFCP@TN after 0.1 mAh cm^{-2} Li deposition.

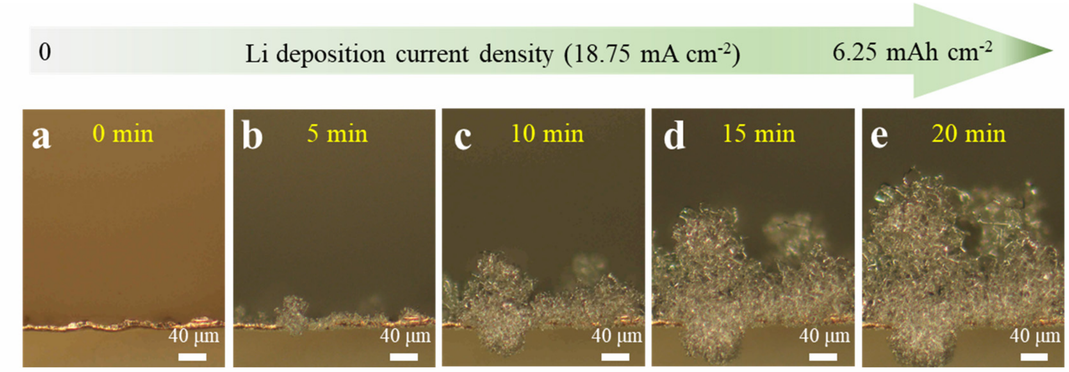

Figure S5. In situ microscopy images of Li deposition behaviors on Cu with various stages.

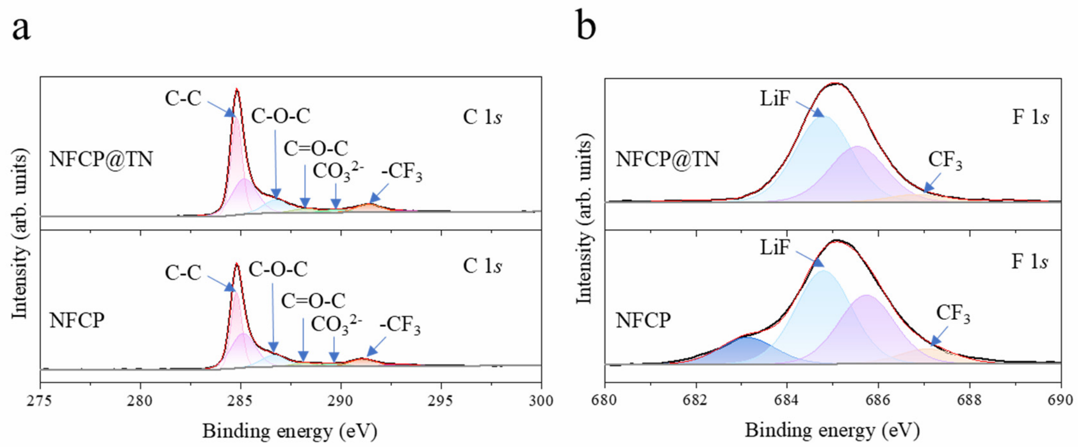

Figure S6. The XPS spectra of NFCP and NFCP@TN electrodes for the (a) C 1*s* spectra, (b) F 1*s* spectra after 0.5 mAh cm^{-2} Li deposition at current density of 0.1 mA cm^{-2}.

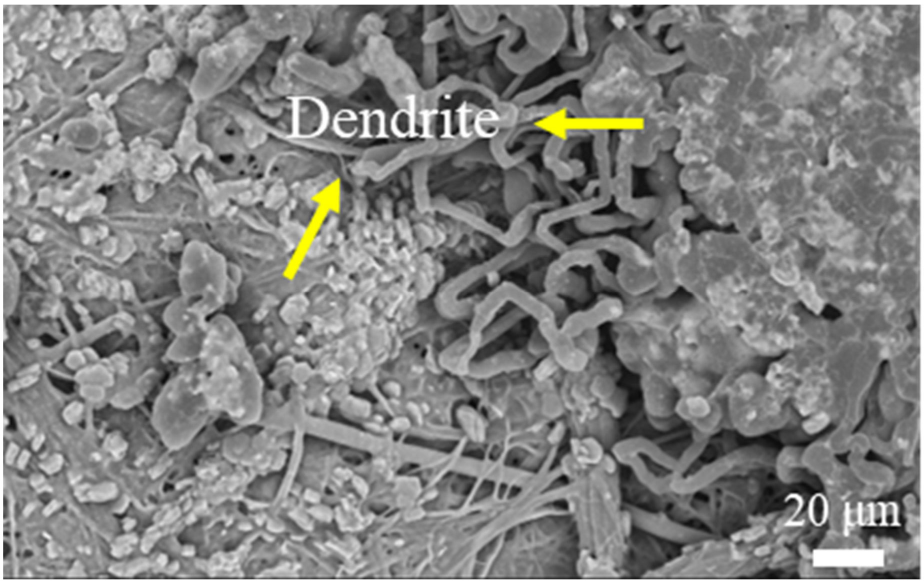

Figure S7. The top view of NFCP after Li/NFCP||NCM811 full cell cycled.

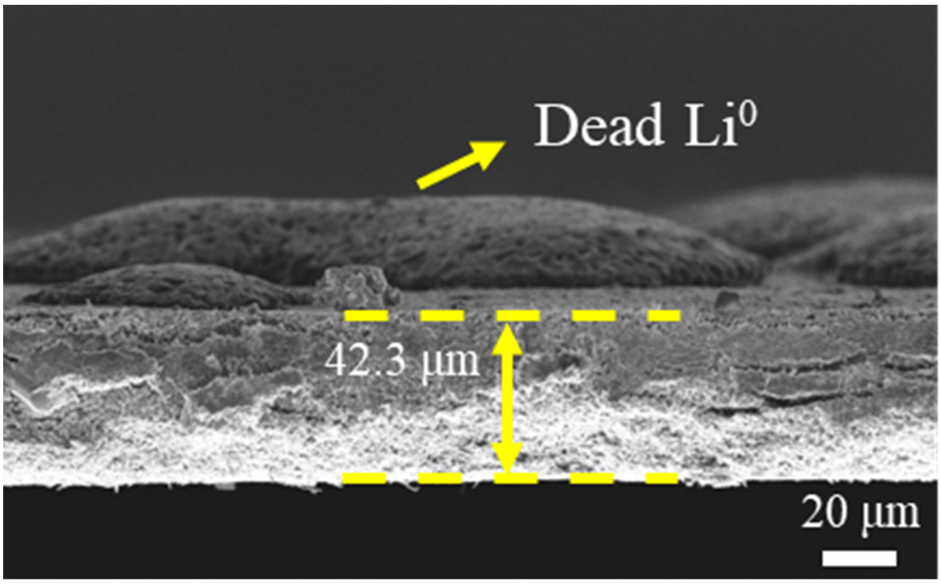

Figure S8. The cross-section of NFCP after Li/NFCP||NCM811 full cell cycled.

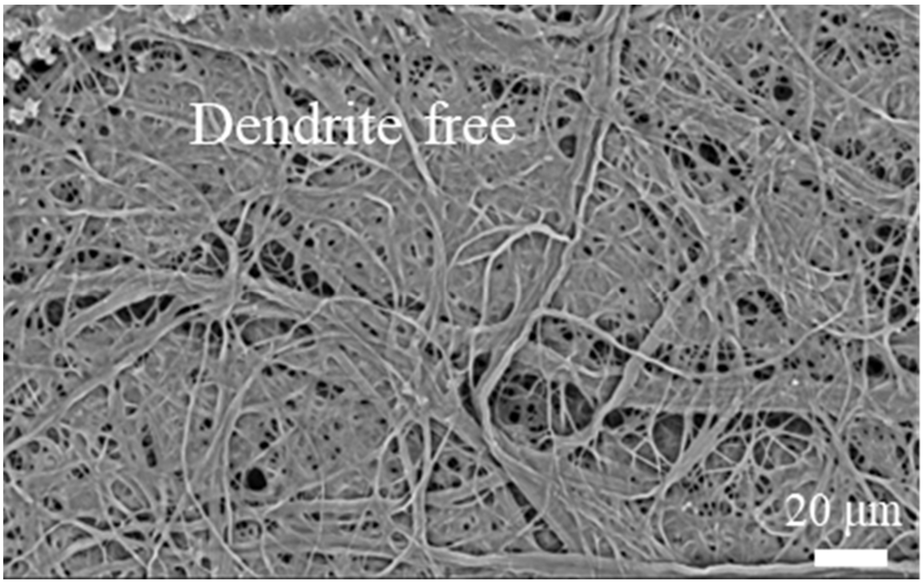

Figure S9. The top view of NFCP@TN after Li/NFCP@TN||NCM811 full cell cycled.

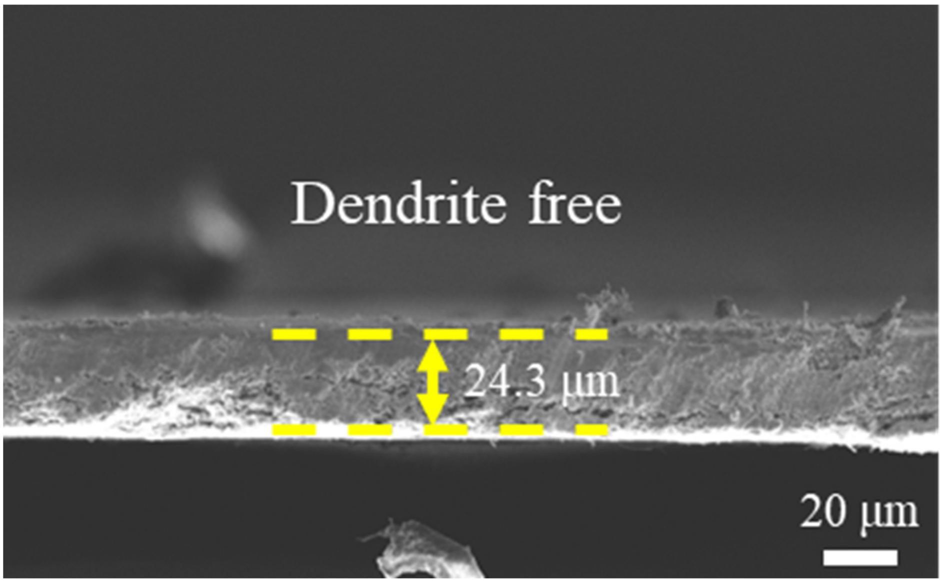

Figure S10. The cross-section of NFCP@TN after Li/NFCP@TN||NCM811 full cell cycled.
